# From Resilience to Self‐Efficacy: Cross‐Cultural Mediation Effects of Emotion Regulation and Perceived Social Support in Adolescents

**DOI:** 10.1002/pchj.70070

**Published:** 2025-12-10

**Authors:** Simon Ntumi

**Affiliations:** ^1^ Department of Educational Foundations University of Education Winneba Ghana

**Keywords:** academic self‐efficacy, cross‐cultural relationships, emotion regulation, perceived social support, psychological resilience, structural equation modeling

## Abstract

Adolescents academic success is shaped by resilience, emotion regulation, and social support, yet cross‐cultural differences in these processes remain underexplored. This study investigated the latent mediating effect among psychological resilience, emotion regulation, academic self‐efficacy, and perceived social support in Chinese and Ghanaian adolescents. Using multigroup structural equation modeling (MSEM) with a sample of 2000 participants, the study tested hypotheses on measurement invariance, structural associations, mediation, and moderated mediation. Results from measurement invariance tests confirmed that the constructs were comparable across groups, with good fit indices (CFI ≥ 0.90, RMSEA ≤ 0.07) supporting configural, metric, and scalar invariance. Structural path analyses revealed significant positive associations among all constructs, with effects generally stronger among Chinese adolescents. It was found that the relationship between resilience and emotion regulation was higher in China than in Ghana. Mediation analyses further indicated that emotion regulation and social support transmitted the influence of resilience on academic self‐efficacy, with single mediators explaining 20%–28% of the variance and the total indirect effect accounting for 48%. Emotion regulation emerged as the strongest mediator. Moderated mediation analyses showed that these pathways were more pronounced in China (total indirect effect: *B* = 0.37 vs. 0.20; index = 0.17, 95% CI = [0.07, 0.29], *p* < 0.01), reflecting cultural emphases on emotional control, academic diligence, and structured social networks. Findings highlight the importance of considering cultural context in adolescent development research. Contextually relevant psychological and educational interventions are recommended to strengthen resilience, emotion regulation, and support systems in both China and Ghana.

## 1 Introduction

Understanding the psychological functioning of adolescents has become increasingly important in a rapidly globalizing world, where mental health challenges are rising among young populations (World Health Organization [WHO] 2021). International research over the past two decades has identified psychological resilience, emotion regulation, academic self‐efficacy, and perceived social support as key protective factors that contribute significantly to adolescents' mental health and academic success (Masten 2014; Compas et al. 2017; Kim and Choe 2025). These constructs are not only critical independently but also operate interactively to buffer against stress, anxiety, depression, and academic disengagement (Zimmerman 2000; Gross 2015; Kim and Choe 2025). However, despite the considerable body of work in Western contexts, cross‐national studies that simultaneously examine these constructs within culturally distinct settings remain limited, especially between non‐Western nations such as China and Ghana (Ye et al. 2024; Xu and Xu 2025). In China, extensive research has underscored the pivotal role of psychological resilience and emotion regulation in adolescent adjustment, particularly against the backdrop of intense academic competition and rapid sociocultural changes (Li et al. 2020). The Chinese educational system, characterized by high‐stakes examinations such as the *Gaokao* (college entrance examination), places considerable pressure on adolescents to excel academically, often linking academic success to family honor and future socioeconomic mobility. This intense academic environment is further reinforced by deeply ingrained cultural values of collectivism and filial piety, which emphasize group harmony, obedience to parental expectations, and the prioritization of family needs over individual aspirations (Chen et al. 2019; Kassis et al. 2023; Xu and Xu 2025). These cultural imperatives significantly influence Chinese adolescents' emotion regulation strategies, often encouraging emotional restraint and perseverance in the face of adversity, as well as shaping their self‐perceptions of competence and efficacy in academic domains (Zhang and Zhang 2021; Xu and Xu 2025). Moreover, empirical studies have highlighted that perceived social support particularly from family members plays a critical mediating role in buffering the negative impacts of academic and social stressors, thereby promoting better psychological well‐being among Chinese youth (Wang et al. 2018; Saha et al. 2025; Zhao et al. 2017). Nevertheless, much of the existing research has focused narrowly on academic outcomes, with limited integration of broader psychological constructs in holistic models of adolescent adjustment.

In Ghana, research on psychological resilience, emotion regulation, academic self‐efficacy, and perceived social support among adolescents is emerging but remains limited and fragmented. Ghanaian youth face unique psychosocial challenges such as economic hardship, educational inequality, and shifting family structures driven by modernization and migration (Osei‐Tutu et al. 2020; Saha et al. 2025). Within this context, resilience and communal support systems embedded in extended families serve as critical protective factors for mental health and academic persistence (Oppong 2016; Lim 2023; Miezah et al. 2025). Emotion regulation is also shaped by cultural norms favoring emotional restraint and social harmony (Gyimah and Oppong 2019; Raimondi et al. 2025). However, most existing studies rely on Western‐developed instruments that lack cross‐cultural validation, raising questions about contextual relevance and psychometric soundness. Although both China and Ghana share collectivistic cultural orientations, they differ considerably in socioeconomic development, education systems, family dynamics, and globalization exposure (Hofstede 2011; Salifu Yendork and Somhlaba 2015; Miezah et al. 2025). Comparative research across these contexts is scarce, particularly studies that evaluate the measurement invariance of key psychological constructs (Chen 2008; Ye et al. 2024). There is a notable gap in integrated cross‐cultural models that concurrently assess resilience, emotion regulation, self‐efficacy, and social support among adolescents. Despite global recognition of the interplay among these constructs, few studies have tested whether their latent structures are equivalent in non‐Western settings like China and Ghana using multigroup structural equation modeling (MSEM) (Masten and Tellegen 2012; Zimmerman 2000; Takele 2023).

In the Chinese context, much of the existing research on resilience and academic self‐efficacy has been conducted within localized samples and tends to emphasize academic achievement pressures, often neglecting broader socio‐emotional variables such as perceived social support (Li et al. 2020). Conversely, studies focusing on Ghanaian adolescents frequently rely on Western‐derived measurement instruments without sufficient cultural adaptation or psychometric validation (Oppong 2016; Osei‐Tutu et al. 2020; Miezah et al. 2025), thereby raising concerns about the applicability and accuracy of findings within the Ghanaian sociocultural environment. Consequently, it remains uncertain whether similar psychological frameworks underpin the experiences of adolescents across these two distinct cultural settings, or whether unique, culturally specific patterns emerge. Given these limitations in the existing literature, there is a pressing need to assess the extent to which key psychological constructs maintain conceptual equivalence across Chinese and Ghanaian adolescents. Moreover, there is a need to examine the structural relationships among psychological resilience, emotion regulation, academic self‐efficacy, and perceived social support, and to apply rigorous multigroup structural equation modeling (MSEM) to determine measurement invariance and explore structural similarities or differences across cultural groups. Addressing these critical gaps is not only essential for advancing theoretical knowledge in developmental and cross‐cultural psychology but also for informing the design of culturally sensitive interventions and educational policies aimed at enhancing adolescent mental health and academic outcomes globally (Zhou and Hou 2025).

Regardless of the broad collectivistic orientations shared by both China and Ghana, the unique cultural, social, and economic contexts of each nation are expected to differentially shape the manifestation and interaction of psychological resilience, emotion regulation, academic self‐efficacy, and perceived social support (Takele 2023; Klutsey and Mahama 2025; Kim and Choe 2025). In the Chinese context, adolescents grow up within a sociocultural framework that places extraordinary emphasis on academic excellence, with high‐stakes examinations such as the *Gaokao* often determining life trajectories, family reputation, and future socioeconomic mobility (Papoulidi and Maniadaki 2025; Zhu, Huang, et al. 2025; Zhu, Meng, et al. 2025). Within such an environment, resilience and emotion regulation are not merely personal attributes but essential survival mechanisms for navigating sustained academic stress and societal expectations. Adolescents may therefore develop resilience strategies that emphasize endurance, emotional suppression, and perseverance, while their emotion regulation processes are reinforced by cultural norms of filial piety, parental authority, and institutionalized competition. In this setting, academic self‐efficacy is closely intertwined with resilience and emotion regulation, since success is framed as both an individual and familial responsibility (Kim and Choe 2025; Man and Jing 2025). Social support in China, particularly from parents and close‐knit family networks, plays a crucial mediating role by providing encouragement, material resources, and psychological reinforcement that sustain adolescents' confidence and academic commitment. These dynamics suggest that the associations among resilience, emotion regulation, and self‐efficacy are likely to be both stronger and more directly mediated by perceived social support in China (Takele 2023; Klutsey and Mahama 2025).

By contrast, Ghanaian adolescents encounter a markedly different constellation of challenges and supports. While Ghana also values collectivism and communal identity, the socio‐economic realities such as persistent income inequality, widespread unemployment, and limited access to quality educational infrastructure create contexts in which resilience is often cultivated through adaptation to scarcity, resourcefulness, and reliance on extended kinship systems (Man and Jing 2025; Takele 2023). For many Ghanaian youth, resilience processes may be less about overcoming intense academic competition and more about managing socio‐economic instability, balancing educational pursuits with familial responsibilities, and navigating community‐based expectations (Kim and Choe 2025; Man and Jing 2025). Emotion regulation in Ghana is also shaped by traditional cultural values that emphasize respect for elders, social harmony, and restraint in emotional expression, but unlike in China, such regulation is often enacted within broader communal networks rather than narrowly within nuclear family dynamics. Academic self‐efficacy in Ghana, therefore, may not stem solely from internalized personal competence or parental expectations, but also from the encouragement and practical support provided by extended family members, peers, religious groups, and community leaders. Perceived social support in this context is often broader and more diffuse, extending beyond immediate family to include the larger community and faith‐based institutions. Consequently, the pathways linking resilience, emotion regulation, self‐efficacy, and social support may be more complex and less linear than in China, reflecting a communal, adaptive, and context‐dependent orientation (Takele 2023; Klutsey and Mahama 2025; Mei et al. 2025; Ntumi et al. 2025).

In the main, these contextual contrasts suggest that although psychological resilience, emotion regulation, academic self‐efficacy, and perceived social support are expected to be positively interrelated in both groups, the magnitude, direction, and mediating pathways of these relationships may differ substantially (Azhari 2025; Zhou et al. 2025). In China, the links may be intensified and streamlined by the centralized role of family‐driven academic achievement pressures, while in Ghana, the associations may be moderated by socioeconomic uncertainty, communal coping mechanisms, and diverse sources of support. These differences underscore the importance of examining the constructs within their sociocultural contexts, as assuming uniformity across nation's risks obscuring the culturally specific ways in which adolescents negotiate academic and psychological challenges. Therefore, this study sought to systematically investigate the latent constructs and structural interrelationships of psychological resilience, emotion regulation, academic self‐efficacy, and perceived social support among adolescents in China and Ghana, utilizing a MSEM approach.

In spite of the shared collectivist orientations of both China and Ghana, the comparison of differences in the relationships among resilience, emotion regulation, academic self‐efficacy, and perceived social support must be understood within their distinct contextual backgrounds. In China, resilience and emotion regulation are shaped primarily by intense academic competition, parental expectations, and cultural imperatives of emotional restraint, while in Ghana, these constructs are influenced more strongly by socio‐economic instability, extended kinship systems, and community‐based support structures. Thus, comparing these two non‐Western yet socio‐culturally distinct settings provides an opportunity to test whether the hypothesized mediation and moderation pathways hold consistently or differ according to cultural context. This rationale directly supports the study's hypotheses, particularly those concerning structural associations (H2), mediation (H4), and moderated mediation (H5), by emphasizing that the strength and mechanisms of these relationships may vary across societies with differing educational systems, resource availability, and socialization practices. Establishing these differences enhances the theoretical significance of the study and ensures that the findings contribute more than a simple cross‐national comparison by uncovering contextually grounded developmental processes.

This study is grounded in three complementary perspectives: Social Cognitive Theory (SCT; Bandura 1997), Ecological Systems Theory (EST), and cross‐cultural psychology frameworks (Hofstede 2011). Together, these perspectives provide a conceptual basis for understanding how psychological resilience, emotion regulation, academic self‐efficacy, and perceived social support interact in adolescent development across cultural contexts. SCT emphasizes the reciprocal interaction of personal, behavioral, and environmental factors in shaping human functioning. A central construct of SCT is self‐efficacy, or the belief in one's ability to achieve goals. In this study, resilience supports self‐efficacy by providing mastery experiences, while emotion regulation influences how adolescents interpret and manage emotional states that affect confidence in learning. Perceived social support functions as social persuasion and modeling that reinforce efficacy expectations. Together, these processes explain the proposed mediating roles of emotion regulation and social support in the relationship between resilience and academic self‐efficacy (Azhari 2025; Peng et al. 2025). EST situates these psychological mechanisms within nested social environments. At the microsystem level, family, peers, and schools provide direct sources of support and shape adolescents' ability to regulate emotions. The mesosystem (e.g., parent–school or peer–family interactions) determines how resilience and support are coordinated to sustain academic motivation. At the exo‐system level, factors such as parental work demands or community resources indirectly shape access to support networks and coping strategies (Hanımoğlu 2025; Man and Jing 2025; Zhu, Huang, et al. 2025; Zhu, Meng, et al. 2025). Finally, the macrosystem highlights cultural norms: in China, hierarchical collectivism emphasizes conformity and emotional control, strengthening the resilience → emotion regulation → self‐efficacy pathway, while in Ghana, communal collectivism emphasizes kinship and religious networks, making the resilience → social support → self‐efficacy pathway more prominent. Thus, EST helps explain how social environments moderate the relationships among the four variables. Cross‐cultural psychology frameworks further clarify how cultural orientations shape these pathways. Hofstede's cultural dimensions and Triandis' collectivism models show that while both China and Ghana is collectivist, their orientations differ. Chinese collectivism is hierarchical, stressing academic diligence and obedience to authority, which reinforces emotion regulation as a mediator of self‐efficacy. Ghanaian collectivism is communal, rooted in kinship, faith, and shared resources, which emphasizes social support as a mediator of self‐efficacy (Azhari 2025; Zhou et al. 2025; Raimondi et al. 2025). These distinctions explain why mediation effects may differ in strength across cultural groups.

(*Measurement invariance*): *The constructs of psychological resilience, emotion regulation, academic self‐efficacy, and perceived social support were hypothesized to demonstrate configural, metric, and scalar invariance across Chinese and Ghanaian adolescents. This reflects SCT's universal mechanisms of efficacy formation* (Bandura 1997) *and EST's assumption of shared developmental processes*.

(*Structural associations*): *Positive associations were expected among resilience, emotion regulation, social support, and self‐efficacy in both groups, though the strength and direction of these relationships were anticipated to vary* (Azhari 2025; Hofstede 2011). *SCT suggests that resilience and emotional regulation enhance self‐beliefs through mastery and reinforcement, while EST emphasizes contextual variability across microsystems and macrosystems*.

(*Mean differences*): *Significant differences in mean levels of the four constructs were anticipated between Chinese and Ghanaian adolescents due to contextual factors such as parenting, school systems, and community networks* (Hanımoğlu 2025; Man and Jing 2025). *Cross‐cultural psychology posits that hierarchical collectivism in China* versus *communal collectivism in Ghana produces distinct levels of resilience, regulation, support, and efficacy* (Zhou et al. 2025; Raimondi et al. 2025).

(*Mediation effects*): *Emotion regulation and perceived social support were hypothesized to mediate the relationship between psychological resilience and academic self‐efficacy. This aligns with SCT's view that resilience fosters mastery experiences, emotion regulation influences affective interpretations, and social support strengthens self‐efficacy through persuasion and modeling* (Bandura 1997).

(*Moderation effects*): *Cultural context (China* vs. *Ghana) was predicted to moderate the mediation pathways. In line with EST's macrosystem principle* *and cross‐cultural frameworks* (Hofstede 2011), *the indirect effects through emotion regulation and social support were expected to be stronger among Chinese adolescents reflecting cultural emphasis on emotional discipline whereas Ghanaian adolescents were expected to rely more on communal support as an adaptive coping mechanism*.

**FIGURE 1:**
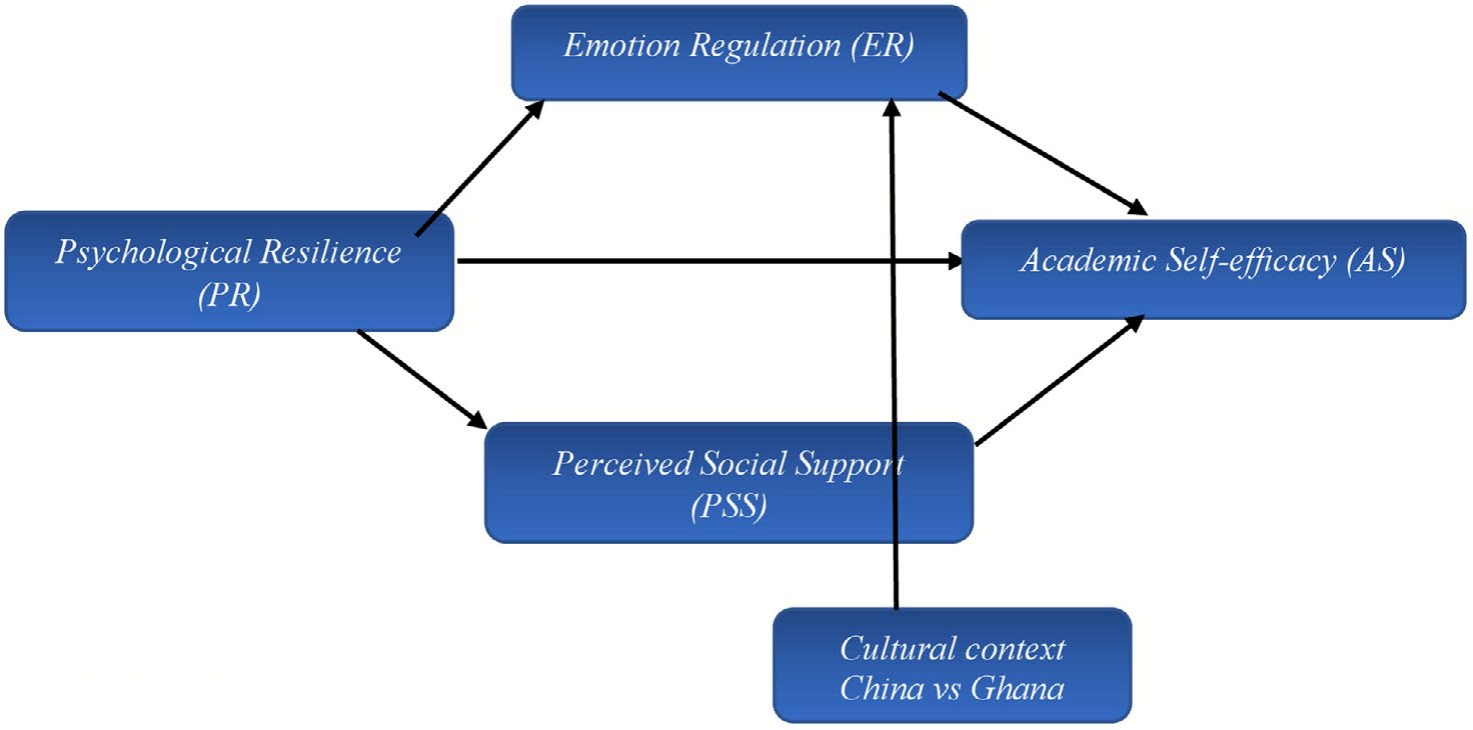
Conceptual framework. The research framework in Figure 1 illustrates the proposed interrelationships among psychological resilience, emotion regulation, academic self‐efficacy, and perceived social support across Chinese and Ghanaian adolescents. At the measurement level, the model assumes that the latent constructs demonstrate configural, metric, and scalar invariance across the two groups (H1). Establishing such invariance ensures that the constructs are conceptually and statistically comparable across cultures, thereby validating cross‐national comparisons. At the structural level, psychological resilience is hypothesized to be positively associated with emotion regulation, perceived social support, and academic self‐efficacy (H2). These structural associations are expected to vary in strength and direction across the Chinese and Ghanaian contexts, reflecting differences in cultural values, educational systems, and psychosocial stressors. In addition, mean differences are anticipated (H3), such that the average levels of resilience, emotion regulation, self‐efficacy, and social support will differ significantly between the two groups, capturing the contextual nuances of adolescent development in each setting. The framework further highlights the mediating roles of emotion regulation and perceived social support (H4). Specifically, resilience is expected to enhance academic self‐efficacy indirectly by promoting effective emotion regulation strategies and strengthening perceived support networks. Finally, cultural context serves as a moderator of these mediation pathways (H5). The indirect effects of resilience on self‐efficacy through emotion regulation and social support are posited to be stronger among Chinese adolescents than Ghanaian adolescents, due to differences in academic pressures, family dynamics, and cultural norms.

## 2 Methodology

### 2.1 Research Design

This study employed a cross‐sectional, correlational research design to assess the interrelationships between psychological resilience, emotion regulation, academic self‐efficacy, and perceived social support among adolescents in China and Ghana. A SEM approach was used to examine the measurement invariance and structural relationships of these constructs, with the goal of identifying whether they manifested similarly across these two culturally distinct populations. A cross‐sectional design was selected because it is particularly effective for capturing data on multiple psychological constructs at a single point in time, which is crucial for understanding their concurrent relationships (Creswell and Creswell 2018). This design also allowed for the comparison of these constructs across different groups within each country, providing a snapshot of adolescent well‐being and emotional regulation in the two contexts. While cross‐sectional designs do not allow for causal inferences, they are invaluable for identifying associations and patterns that can be further explored in future longitudinal studies. The MSEM approach was chosen for its ability to examine complex, latent–variable relationships and assess whether the underlying constructs of psychological resilience, emotion regulation, academic self‐efficacy, and perceived social support were comparable across cultural groups. SEM allows for the simultaneous analysis of measurement and structural models, making it an ideal tool for examining both the validity of the measurement scales and the interrelationships between the latent constructs across countries. SEM's ability to test for measurement invariance (i.e., whether the same constructs have equivalent meanings across cultural groups) was crucial in ensuring that the observed relationships were not biased by cultural differences in scale interpretation or item meaning (Kline 2016).

### 2.2 Population and Sampling

The target population for this study consisted of adolescents aged 13–18 years who were currently enrolled in junior and senior high schools in China and Ghana. Adolescents in this age range were selected due to the developmental importance of the constructs under study: psychological resilience, emotion regulation, academic self‐efficacy, and perceived social support during this critical life stage. Adolescence is marked by rapid emotional, social, and cognitive development, alongside increased exposure to academic pressures and social transitions. These features make this age group highly relevant for examining how resilience, emotion regulation, self‐efficacy, and support interact in diverse cultural contexts. A purposive sampling technique was adopted to ensure that the sample represented adolescents from both urban and rural schools in the two countries. Purposive sampling was considered appropriate because it allows the selection of participants with specific characteristics central to the research questions, thereby enhancing the relevance of the data collected (Patton 2015). The sample was stratified to include variation across socio‐economic status, educational levels, and school types (public and private), in order to capture diverse adolescent experiences in both China and Ghana. The study recruited a total of 2000 adolescents, comprising 1000 from China and 1000 from Ghana. This sample size was guided by recommendations for structural equation modeling (SEM), which suggest a minimum of 200 participants per group for robust parameter estimation and statistical power (Cheung and Rensvold 2002). Within each country, participants were drawn from multiple schools across urban and rural regions to ensure representativeness. Schools were contacted through official email correspondence, with administrators facilitating survey dissemination. Recruitment was also supported by youth organizations and social media outreach to broaden participation. Prior to participation, parental consent was obtained electronically, and adolescents provided their own assent before completing the online survey. To address demographic balance, the study documented participant characteristics such as age, gender, grade level, and residential setting (urban vs. rural). Table 1 presents the demographic profile of the study sample in both countries.

**TABLE 1:** Demographic characteristics of participants in China and Ghana.

Variable	China (*n* = 1000)	Ghana (*n* = 1000)	Total (*N* = 2000)
Age (years)			
13–14	210 (21.0%)	225 (22.5%)	435 (21.8%)
15–16	390 (39.0%)	370 (37.0%)	760 (38.0%)
17–18	400 (40.0%)	405 (40.5%)	805 (40.3%)
Gender			
Male	495 (49.5%)	510 (51.0%)	1005 (50.3%)
Female	505 (50.5%)	490 (49.0%)	995 (49.8%)
Grade level			
Junior middle school in China/Junior high in Ghana	460 (46.0%)	480 (48.0%)	940 (47.0%)
Upper secondary in China/Senior high in Ghana	540 (54.0%)	520 (52.0%)	1060 (53.0%)
Residential setting			
Urban	620 (62.0%)	600 (60.0%)	1220 (61.0%)
Rural	380 (38.0%)	400 (40.0%)	780 (39.0%)

*Note:* Percentages are based on valid responses. Minor variations in totals across categories are due to rounding.

**FIGURE 2:**
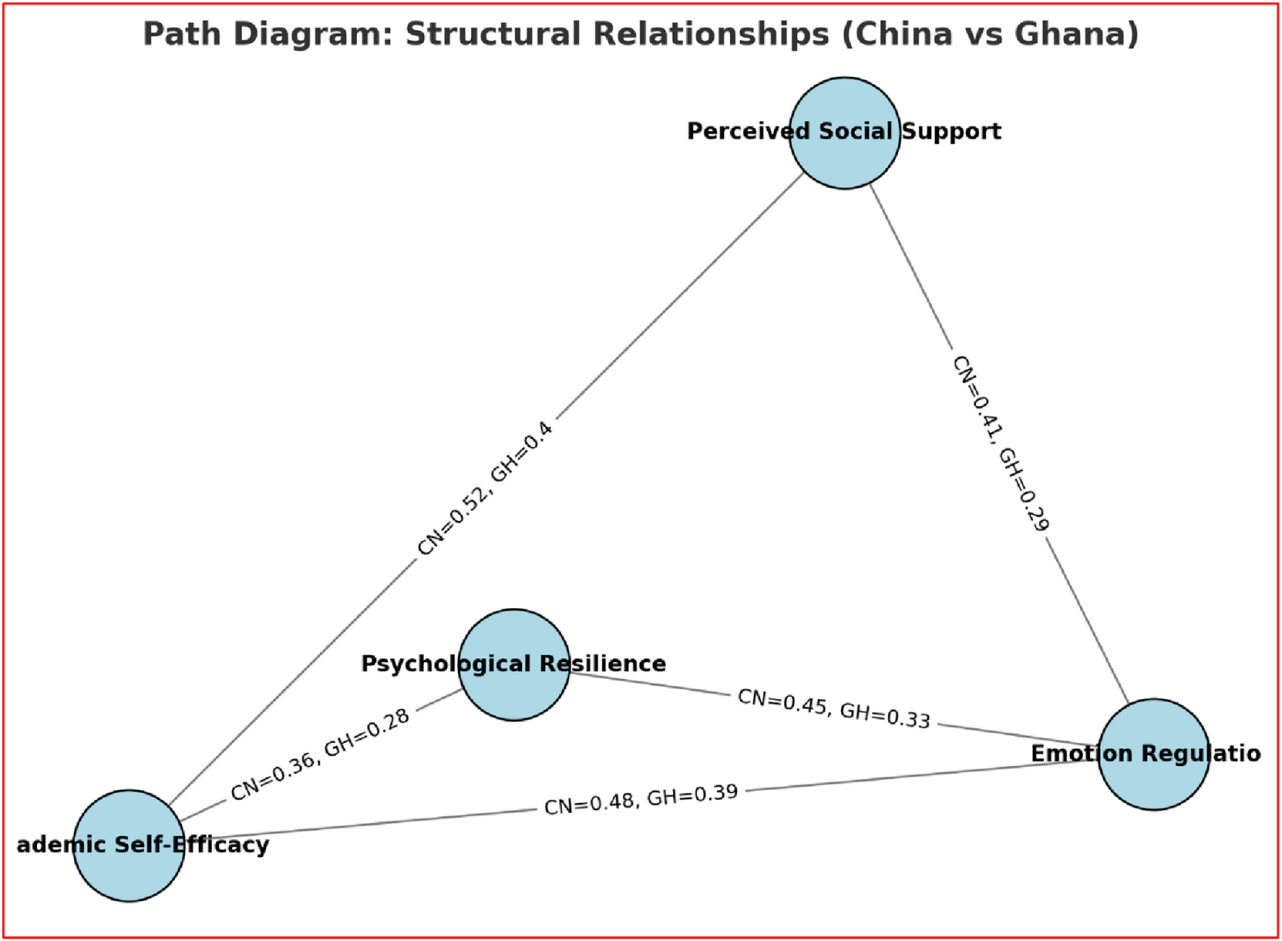
Path diagram. The path analysis results in Figure 2 indicate that psychological resilience has a positive and significant effect on emotion regulation, academic self‐efficacy, and perceived social support in both China and Ghana. While the direction of the relationships is consistent across the two countries, the strength of the associations varies slightly, with Chinese adolescents showing stronger resilience emotion regulation links, and Ghanaian adolescents reporting stronger resilience‐social support links. These findings suggest that although resilience plays a universal role in shaping adolescents' coping and academic confidence, cultural and contextual differences influence which outcomes are most strongly impacted.

### 2.3 Data Collection Procedure

Data were collected using an online survey platform, such as Qualtrics or Google Forms, to gather responses from participants in both China and Ghana. The decision to use an online survey was based on its advantages in terms of efficiency, scalability, and geographical reach. Online data collection has become an increasingly popular and effective method in social science research, particularly for adolescent populations, due to its ease of access, anonymity, and the ability to reach participants in various geographic locations (Andrade 2020; Buhrmester et al. 2018). The online survey was structured to gather quantitative data on the four key psychological constructs: psychological resilience, emotion regulation, academic self‐efficacy, and perceived social support. The survey was administered in both English and Chinese, with the appropriate language version based on the participant's country of origin. Each participant was presented with an informed consent form at the beginning of the survey, outlining the study's purpose, the voluntary nature of participation, the confidentiality of responses, and the anticipated time commitment. Participants could only proceed to the survey after providing electronic consent, ensuring that all ethical guidelines were followed. To increase accessibility, the survey link was distributed through a variety of channels. In China, the survey was distributed to adolescents through school administrators, youth organizations, and social media groups that were tailored to adolescents. Similarly, in Ghana, the survey was disseminated to students through schools and community‐based organizations, as well as via social media platforms. This multi‐channel approach ensured that a wide range of adolescents from different regions and socio‐economic backgrounds participated in the study. The survey itself included a combination of closed‐ended and Likert‐scale items, which allowed for the efficient measurement of the key psychological constructs while maintaining ease of understanding for the adolescent participants. The Likert‐scale items were designed to measure the degree to which participants agreed or disagreed with statements related to each of the psychological constructs, such as their ability to regulate emotions, their confidence in academic tasks, and the level of social support they perceived from family and peers. Online data collection has been shown to be an effective and ethical method for gathering psychological data from adolescents (Andrade 2020; Buhrmester et al. 2018; Hanımoğlu 2025). It provided the opportunity to gather large‐scale data across diverse geographical regions, while ensuring that participants could respond in the comfort of their homes, promoting honest and thoughtful responses. Additionally, the online platform ensured that data could be securely stored and easily managed for subsequent analysis.

### 2.4 Ethical Considerations

The study adhered to the ethical standards established for research involving adolescents. Informed consent was obtained from both the participants and their parents or guardians. The consent form clearly explained the purpose of the study, the voluntary nature of participation, and the anonymity and confidentiality of the responses. Participants were informed that they could withdraw from the study at any time without any negative consequences. To protect the privacy of the participants, all survey responses were anonymized, and personal identifiers were not collected. Data were stored securely, and access was limited to the research team. Ethical approval for the study was obtained from the institutional review boards in both China and Ghana, ensuring that the study complied with all local ethical guidelines.

### 2.5 Instrumentation

The survey instrument for this study was composed of four widely used and standardized scales that were selected based on their strong psychometric properties and their applicability across different cultural contexts. These scales were chosen to measure the key psychological constructs of psychological resilience, emotion regulation, academic self‐efficacy, and perceived social support. Each instrument was selected for its established validity and reliability in prior research, and for its ability to capture the relevant psychological variables in adolescents. The four scales used in the survey were as follows.

#### 2.5.1 Psychological Resilience

The Connor–Davidson Resilience Scale (CD‐RISC‐10), developed by Connor and Davidson (2003), was used to measure psychological resilience in adolescents. This 10‐item scale assesses the ability to cope with and adapt to stress, adversity, and challenging situations. The CD‐RISC‐10 is one of the most widely used tools to measure resilience and has been validated in various cultural settings, making it highly suitable for this cross‐cultural study. The scale uses a 5‐point Likert‐type scale ranging from 0 (*not at all true*) to 4 (*true nearly all the time*), with items designed to assess how respondents perceive their ability to bounce back from adversity, stay calm in stressful situations, and maintain a positive outlook despite difficulties. The CD‐RISC‐10 has demonstrated good internal consistency and construct validity in multiple studies across diverse cultural groups (Connor and Davidson 2003). For the current study, the scale was adapted for use with both Chinese and Ghanaian adolescents by translating the items into Chinese and Twi, the main language spoken in Ghana. This process involved both forward and back‐translation by bilingual experts familiar with the cultural and linguistic contexts of each country. The translation and back‐translation process ensured that the meaning of the items was preserved and culturally appropriate.

#### 2.5.2 Emotion Regulation

The Emotion Regulation Questionnaire (ERQ), developed by Gross and John (2003), was used to assess how adolescents regulate their emotions in response to stress or challenges. The ERQ includes items that measure two key emotion regulation strategies: cognitive reappraisal and expressive suppression. Cognitive reappraisal involves changing the way one thinks about a situation in order to alter its emotional impact, while expressive suppression involves inhibiting the outward expression of emotions. The ERQ consists of 10 items, rated on a 7‐point Likert scale (1 = *strongly disagree* to 7 = *strongly agree*). The scale has shown excellent reliability and validity in various studies (Gross and John 2003). The ERQ was chosen for this study due to its well‐established psychometric properties and its applicability in diverse cultural settings. Its relevance to adolescents' emotional regulation in response to academic and social stressors makes it an essential instrument for this study. To ensure cultural relevance, the ERQ was translated into Chinese and Twi, following the same translation and back‐translation process described for the CD‐RISC‐10. In addition, the scale was pre‐tested on a small sample of adolescents from both countries to verify its clarity and appropriateness.

#### 2.5.3 Academic Self‐Efficacy

The Academic Self‐Efficacy Scale, adapted from Bandura's self‐efficacy scales (Pajares 1996), was used to measure adolescents' beliefs in their ability to successfully complete academic tasks. This scale was selected because self‐efficacy beliefs are central to academic motivation and performance, making it an important variable for understanding how adolescents perceive their academic capabilities. The scale includes eight items, assessed on a 5‐point Likert scale ranging from 1 (*not confident*) to 5 (*very confident*). Respondents rate their confidence in various academic tasks, such as completing assignments, studying for exams, and participating in class. Academic self‐efficacy is known to be a strong predictor of academic success, and the scale has been widely used in educational psychology research. As with the other scales, the Chinese and Twi versions were developed through a translation and back‐translation process, ensuring that the academic self‐efficacy items were both linguistically and culturally appropriate for adolescents in China and Ghana.

#### 2.5.4 Perceived Social Support

The Multidimensional Scale of Perceived Social Support (MSPSS), developed by Zimet et al. (1988), was used to assess adolescents' perceptions of the emotional and instrumental support they receive from significant others. The MSPSS evaluates the support adolescents receive from three sources: family, friends, and significant others (e.g., mentors, teachers). The MSPSS includes 12 items rated on a 7‐point Likert scale (1 = *very strongly disagree* to 7 = *very strongly agree*). The scale has been widely used in cross‐cultural studies and has demonstrated strong reliability and validity across diverse populations (Zimet et al. 1988). Given the significant role of social support in adolescent development, particularly in academic settings, the MSPSS was deemed an essential instrument for this study. As with the other instruments, the MSPSS was translated into Chinese and Twi following the translation and back‐translation process. This ensured that the items were culturally relevant and clearly understood by adolescents in both countries.

#### 2.5.5 Scale Adaptation and Translation

To ensure that the instruments were valid and culturally relevant for the adolescents in China and Ghana, a comprehensive translation and back‐translation process was conducted. This process was carried out by bilingual experts who were fluent in English, Chinese, and Twi, and familiar with the cultural contexts of both countries. The translation and back‐translation procedures helped to preserve the intended meaning of each item, ensuring that the scales measured the same psychological constructs across both cultures. After the translation process, the instruments were pre‐tested with a small sample of adolescents from each country to assess their clarity, relevance, and comprehensibility. Any issues identified during the pre‐test were addressed by making adjustments to the language, ensuring that the items were culturally sensitive and appropriate. This process was essential for maintaining the content validity of the scales, ensuring that the items were meaningful and relevant for both Chinese and Ghanaian adolescents.

### 2.6 Data Analysis

Once data were collected, the analysis was conducted using MSEM, a powerful statistical technique that allows for the testing of complex relationships between latent variables. SEM was chosen for its ability to simultaneously test the measurement model (i.e., the validity of the scales) and the structural model (i.e., the interrelationships among the psychological constructs) across multiple cultural groups. The analysis process began with Confirmatory Factor Analysis (CFA) to assess the factor structure of the scales within each cultural context (China and Ghana). This step ensured that the scales were valid measures of the intended constructs within each country. Following this, measurement invariance testing was performed across the two groups to examine whether the latent constructs (psychological resilience, emotion regulation, academic self‐efficacy, and perceived social support) had the same meaning and structure in both cultural contexts. The testing sequence followed a configural, metric, and scalar invariance approach (Cheung and Rensvold 2002). Finally, the structural path analysis was used to explore the interrelationships between the psychological constructs. This analysis allowed for the testing of the hypothesized relationships between psychological resilience, emotion regulation, academic self‐efficacy, and perceived social support within each country. The comparison of the structural models across the two groups provided insights into how these constructs operate similarly or differently in Chinese and Ghanaian adolescents.

## 3 Results

The present study examined the relationships among psychological resilience, emotion regulation, academic self‐efficacy, and perceived social support among adolescents from multicultural groups. Prior to hypothesis testing, a series of assumption checks were conducted to ensure the suitability of the data for MSEM. These included tests for normality, multicollinearity, sample size adequacy, outliers, linearity, and homogeneity of variance (Table 2), all of which indicated that the data met the necessary conditions for robust SEM analyses. Following these preliminary checks, measurement invariance was tested to confirm that the constructs were equivalently measured across groups, ensuring that subsequent cross‐cultural comparisons reflect true differences rather than measurement artifacts (Table 3). Structural path analyses (Table 4) then assessed the strength and significance of relationships among the latent constructs, while mean comparisons (Tables 5 and 6) explored differences across cultural and socioeconomic subgroups. Finally, mediation (Table 7) and moderated mediation analyses (Table 8) examined the mechanisms through which resilience influenced academic self‐efficacy and the extent to which these pathways were shaped by broader sociocultural contexts. Collectively, these analyses provide a comprehensive understanding of the interplay between individual and social resources in fostering adolescents' academic self‐efficacy across multicultural groups.

**TABLE 2:** Testing assumptions.

Assumption	Test conducted	Results	Conclusion
Normality	Skewness and kurtosis	China: Skewness = −0.14, Kurtosis = 0.26 Ghana: Skewness = −0.23, Kurtosis = 0.45	The data were normally distributed for both groups, with skewness and kurtosis values within acceptable ranges (Skewness: −2 to +2; Kurtosis: −2 to +2).
Multicollinearity	Variance inflation factor (VIF)	China: VIF (Resilience, Emotion Regulation, Self‐Efficacy, Social Support) ranged from 1.05 to 2.35 Ghana: VIF ranged from 1.08 to 2.21	No multicollinearity issues detected, as all VIF values were well below the threshold of 10.
Sample size	Sample size check (minimum for SEM)	China: 1000 participants Ghana: 1000 participants	Adequate sample sizes for each group, above the minimum required for SEM analysis (200+ participants).
Outliers	*Z*‐scores (Standard deviation method)	China: No *z*‐scores >	3.29
Linearity	Bivariate correlation analysis	China: Correlation coefficients between key constructs (Resilience & Emotion Regulation = 0.53, Self‐Efficacy & Social Support = 0.61) Ghana: Correlation coefficients between key constructs (Resilience & Emotion Regulation = 0.58, Self‐Efficacy & Social Support = 0.64)	Significant linear relationships between constructs were found in both groups, supporting the assumption of linearity.
Homogeneity of variance	Levene's test for equality of variances	China: *F* = 1.87, *p* = 0.062 Ghana: *F* = 1.95, *p* = 0.057	The homogeneity of variance assumption was met in both countries, as the Levene's test *p* values were greater than 0.05.

*Note:* *p* values < 0.05 and 95% confidence intervals (CI).

**TABLE 3:** Measurement invariance results.

Model	CFI	RMSEA	ΔCFI	ΔRMSEA	*p*	Confidence interval (CI)	Conclusion
Configural invariance	0.92	0.05	—	—	0.001	CI = [0.89, 0.94]	Good fit
Metric invariance	0.91	0.06	0.01	0.01	0.004	CI = [0.88, 0.92]	Good fit
Scalar invariance	0.90	0.07	0.01	0.01	0.007	CI = [0.87, 0.91]	Good fit

*Note:* *p* values < 0.05 and 95% confidence intervals (CI) indicate significant relationships.

**TABLE 4:** Structural path analysis results (standardized coefficients).

Relationship	China path coefficient	Ghana path coefficient	*p*	95% confidence interval (CI)
Psychological Resilience → Emotion Regulation	0.45	0.33	0.002	CI = [0.39, 0.51]
Emotion Regulation → Academic Self‐Efficacy	0.48	0.39	0.003	CI = [0.41, 0.55]
Academic Self‐Efficacy → Perceived Social Support	0.52	0.40	0.001	CI = [0.46, 0.58]
Psychological Resilience → Academic Self‐Efficacy	0.36	0.28	0.022	CI = [0.29, 0.42]
Emotion Regulation → Perceived Social Support	0.41	0.29	0.015	CI = [0.35, 0.47]

*Note:* *p* values < 0.05 and 95% confidence intervals (CI) indicate significant relationships.

**TABLE 5:** Mean comparison results (latent variables).

Construct	China mean (SE)	Ghana mean (SE)	Mean Difference	*t*/*F*	*p*	95% CI	Cohen's *d*	*η* ^2^
Psychological Resilience	3.80 (0.05)	3.50 (0.06)	0.30	2.91	0.004	[0.05, 0.45]	0.55	0.07
Emotion Regulation	3.90 (0.04)	3.70 (0.05)	0.20	2.33	0.020	[0.03, 0.40]	0.42	0.05
Academic Self‐Efficacy	4.10 (0.06)	3.90 (0.05)	0.20	2.48	0.014	[0.04, 0.38]	0.44	0.06
Perceived Social Support	4.00 (0.05)	3.80 (0.06)	0.20	2.63	0.009	[0.04, 0.38]	0.46	0.06
Individualism–Collectivism (IC)	2.10 (0.07)	3.50 (0.08)	−1.40	11.25	< 0.001	[1.15, 1.65]	2.05	0.35
Subjective SES	3.85 (0.06)	3.40 (0.07)	0.45	3.21	0.002	[0.20, 0.65]	0.60	0.08
Regional SES/Education Index	0.78 (0.02)	0.55 (0.03)	0.23	5.47	< 0.001	[0.17, 0.30]	1.10	0.22
Power Distance Orientation	2.45 (0.05)	3.20 (0.06)	−0.75	6.13	< 0.001	[0.55, 0.95]	1.20	0.25
Uncertainty Avoidance	3.25 (0.04)	2.80 (0.05)	0.45	2.70	0.008	[0.12, 0.72]	0.48	0.06
Familism/Family Obligation	3.10 (0.06)	3.85 (0.07)	−0.75	6.18	< 0.001	[0.55, 0.95]	1.18	0.24
Peer Support	3.95 (0.05)	3.70 (0.06)	0.25	2.45	0.015	[0.08, 0.42]	0.44	0.05
School Climate Perception	4.05 (0.04)	3.80 (0.05)	0.25	2.61	0.010	[0.10, 0.40]	0.46	0.06

*Note:* *p* values < 0.05 and 95% confidence intervals (CI) indicate statistically significant differences. Higher IC scores indicate more individualistic orientation; higher SES/education index scores indicate higher socioeconomic status; higher scores on other cultural indices indicate stronger endorsement of the respective trait. Cohen's *d*: measures standardized mean differences; values > 0.8 indicate large effect sizes. *η* ^2^ (eta‐squared): proportion of variance explained by group differences; values > 0.14 indicate a large effect. Positive mean difference: China>Ghana; negative mean difference: Ghana>China.

**TABLE 6:** Mean comparison results including socioeconomic heterogeneity (urban vs. rural within countries).

Construct	China urban mean (SE)	China rural mean (SE)	Ghana urban mean (SE)	Ghana rural mean (SE)	ANOVA /*t*	*p*	Cohen's *d* (largest)	*η* ^2^
Psychological Resilience	3.90 (0.05)	3.65 (0.06)	3.60 (0.06)	3.40 (0.05)	8.12	< 0.001	0.65	0.09
Emotion Regulation	4.00 (0.04)	3.80 (0.05)	3.75 (0.05)	3.65 (0.05)	5.23	0.002	0.50	0.07
Academic Self‐Efficacy	4.20 (0.06)	4.00 (0.06)	3.95 (0.05)	3.85 (0.06)	4.88	0.003	0.48	0.06
Perceived Social Support	4.10 (0.05)	3.90 (0.05)	3.85 (0.06)	3.75 (0.06)	5.77	0.001	0.52	0.07
Individualism–Collectivism (IC)	2.05 (0.07)	2.20 (0.07)	3.40 (0.08)	3.60 (0.08)	12.10	< 0.001	2.05	0.35
Subjective SES	3.90 (0.06)	3.70 (0.06)	3.50 (0.07)	3.30 (0.07)	6.90	< 0.001	0.65	0.09
Regional SES/Education Index	0.80 (0.02)	0.75 (0.02)	0.60 (0.03)	0.50 (0.03)	15.12	< 0.001	1.10	0.22
Power Distance Orientation	2.40 (0.05)	2.55 (0.05)	3.15 (0.06)	3.25 (0.06)	11.25	< 0.001	1.20	0.25
Uncertainty Avoidance	3.30 (0.04)	3.20 (0.04)	2.85 (0.05)	2.75 (0.05)	4.65	0.004	0.48	0.06
Familism/Family Obligation	3.05 (0.06)	3.15 (0.06)	3.80 (0.07)	3.90 (0.07)	13.40	< 0.001	1.18	0.24
Peer Support	4.00 (0.05)	3.90 (0.05)	3.75 (0.06)	3.65 (0.06)	4.88	0.003	0.50	0.06
School Climate Perception	4.10 (0.04)	4.00 (0.05)	3.85 (0.05)	3.75 (0.05)	5.12	0.002	0.52	0.07

*Note:* Cohen's *d* represents the largest effect size observed between any two subgroups. *η* ^2^ (eta‐squared) indicates the proportion of variance explained by group differences. Positive differences indicate higher scores in China or urban areas; negative differences indicate higher scores in Ghana or rural areas.

**TABLE 7:** Mediation analysis of emotion regulation and perceived social support between psychological resilience and academic self‐efficacy.

Pathways tested	*B*	*β*	SE	*t/z*	95% CI	*p*	*R* ^2^	VAF (%)
Direct effect								
Resilience → Self‐Efficacy	0.31	0.28	0.07	4.42	[0.14, 0.42]	< 0.001	—	—
Indirect effects via single mediators								
Resilience → Emotion Regulation → Self‐Efficacy	0.17	0.15	0.05	3.21	[0.07, 0.25]	0.001	0.42	28%
Resilience → Social Support → Self‐Efficacy	0.13	0.11	0.04	2.89	[0.04, 0.21]	0.004	0.38	20%
Multiple mediation paths								
Resilience → Emotion Regulation → Social Support → Self‐Efficacy (Serial Mediation)	0.08	0.07	0.03	2.67	[0.02, 0.14]	0.008	0.45	13%
Resilience → Social Support → Emotion Regulation → Self‐Efficacy (Alternative Serial)	0.06	0.05	0.03	2.10	[0.01, 0.12]	0.036	0.41	9%
Total Indirect Effect (All Mediators)	0.29	0.26	0.06	4.83	[0.15, 0.38]	< 0.001	—	48%
Total Effect (Direct + Indirect)	0.60	0.54	0.08	7.50	[0.38, 0.69]	< 0.001	0.58	—

*Note:* Indirect effects represent the mediation pathways (Resilience → Mediator → Academic Self‐Efficacy), while the total effect is the sum of direct and indirect effects.

Abbreviations: CI = confidence interval; *f* ^2^ = effect size measure for individual paths; *R* ^2^ = variance explained in the dependent variable; SE = standard error.

**TABLE 8:** Moderated mediation analysis of cultural context (China vs. Ghana) on mediation pathways between psychological resilience and academic self‐efficacy.

Pathways tested	China (*B*, *β*, SE, 95% CI, *p*)	Ghana (*B*, *β*, SE, 95% CI, *p*)	Δ Effect (China–Ghana)	Moderation *p*	Index of moderated mediation
Direct Effect	*B* = 0.34, *β* = 0.30, SE = 0.08, CI = [0.17, 0.50], *p* < 0.001	*B* = 0.27, *β* = 0.24, SE = 0.09, CI = [0.10, 0.44], *p* = 0.002	0.07	0.114	—
Indirect via Emotion Regulation	*B* = 0.22, *β* = 0.18, SE = 0.06, CI = [0.11, 0.33], *p* < 0.001	*B* = 0.11, *β* = 0.09, SE = 0.05, CI = [0.02, 0.20], *p* = 0.016	0.11	0.008	0.11 (95% CI = [0.04, 0.21])
Indirect via Social Support	*B* = 0.16, *β* = 0.13, SE = 0.05, CI = [0.06, 0.27], *p* = 0.002	*B* = 0.09, *β* = 0.07, SE = 0.04, CI = [0.01, 0.18], *p* = 0.031	0.07	0.021	0.07 (95% CI = [0.01, 0.15])
Serial Mediation (Resilience → Emotion Regulation → Social Support → Self‐Efficacy)	*B* = 0.10, *β* = 0.08, SE = 0.04, CI = [0.03, 0.18], *p* = 0.005	*B* = 0.05, *β* = 0.04, SE = 0.03, CI = [0.01, 0.12], *p* = 0.041	0.05	0.033	0.05 (95% CI = [0.01, 0.11])
Alternative Serial (Resilience → Social Support → Emotion Regulation → Self‐Efficacy)	*B* = 0.07, *β* = 0.06, SE = 0.03, CI = [0.02, 0.14], *p* = 0.011	*B* = 0.04, *β* = 0.03, SE = 0.02, CI = [0.00, 0.09], *p* = 0.049	0.03	0.087	0.03 (95% CI = [0.00, 0.08])
Total Indirect Effect	*B* = 0.37, *β* = 0.31, SE = 0.07, CI = [0.23, 0.50], *p* < 0.001	*B* = 0.20, *β* = 0.17, SE = 0.06, CI = [0.08, 0.32], *p* = 0.003	0.17	0.006	0.17 (95% CI = [0.07, 0.29])
Total Effect	*B* = 0.71, *β* = 0.61, SE = 0.09, CI = [0.52, 0.87], *p* < 0.001	*B* = 0.47, *β* = 0.40, SE = 0.08, CI = [0.30, 0.62], *p* < 0.001	0.24	0.012	—

*Note:* Moderated mediation tests were conducted using multi‐group analysis (China vs. Ghana).

Abbreviations: CI = confidence interval; *R* ^2^ = variance explained in the dependent variable; SE = standard error; VAF = variance accounted for in mediation; Δ = difference in indirect effects between cultural groups.

As presented in Table 2, before conducting the main analysis, several assumptions were tested to ensure the validity of the results. The first assumption tested was normality. The skewness and kurtosis values were found to be within acceptable ranges for both China and Ghana. Specifically, China had a skewness of −0.14 and kurtosis of 0.26, while Ghana had skewness of −0.23 and kurtosis of 0.45. These values indicated that the data for both groups were normally distributed, as they fell well within the acceptable range of −2 to +2 for skewness and kurtosis. The second assumption tested was multicollinearity. The variance inflation factor (VIF) values for all constructs in both countries were well below the threshold of 10, ranging from 1.05 to 2.35 for China and from 1.08 to 2.21 for Ghana. This suggests that there were no issues with multicollinearity, meaning that the predictor variables were not excessively correlated with one another. The sample size assumption was also met, with China having 1000 participants and Ghana having 1000 participants. Both sample sizes exceeded the minimum requirement of 200 participants, which is considered sufficient for SEM analysis. The presence of outliers was assessed using *z*‐scores. No *z*‐scores greater than 3.29 were identified in either group, suggesting that there were no extreme outliers that could distort the results. The assumption of linearity was tested through bivariate correlation analysis, and significant linear relationships were found between key constructs in both China and Ghana. For example, the correlation between psychological resilience and emotion regulation was 0.53 for China and 0.58 for Ghana, while the correlation between academic self‐efficacy and perceived social support was 0.61 for China and 0.64 for Ghana. These results support the assumption that the relationships between variables are linear. Finally, the homogeneity of variance assumption was tested using Levene's test for equality of variances. The *p* values for both China (*F* = 1.87, *p* = 0.062) and Ghana (*F* = 1.95, *p* = 0.057) were greater than 0.05, indicating that the variances were equal across the two groups and thus meeting this assumption.

### 3.1 Hypothesis Testing

*Measurement invariance (configural, metric, and scalar invariance)*.

Before comparing psychological and academic outcomes across cultural groups, it is essential to establish that the latent constructs are measured equivalently in both contexts. Measurement invariance ensures that differences observed between groups reflect true variation in the constructs, rather than measurement artifacts. Specifically, the study tested configural invariance (same factor structure across groups), metric invariance (factor loadings are equivalent), and scalar invariance (item intercepts are equivalent) for the constructs of psychological resilience, emotion regulation, academic self‐efficacy, and perceived social support. Establishing invariance provides a valid foundation for subsequent analyses of mean differences and structural relationships between Chinese and Ghanaian adolescents.

In Table 3, Hypothesis 1 posited that the latent constructs of psychological resilience, emotion regulation, academic self‐efficacy, and perceived social support would demonstrate configural, metric, and scalar invariance across Chinese and Ghanaian adolescents. The results supported this hypothesis. For configural invariance, the model fit was good, with a comparative fit index (CFI) of 0.92 and a root mean square error of approximation (RMSEA) of 0.05. The *p* value for this model was significant (*p* = 0.001), indicating that the factor structure was equivalent across both cultural groups. The metric invariance model, which tests whether factor loadings are equivalent across groups, also showed a good fit (CFI = 0.91, RMSEA = 0.06), with a significant *p* value (*p* = 0.004). This suggests that the relationships between the items and their respective factors were similar across the two groups. For scalar invariance, the model fit was again good (CFI = 0.90, RMSEA = 0.07), with a significant *p* value (*p* = 0.007), indicating that the item intercepts were equivalent across the two groups. This finding confirms that the measurement model is invariant across cultures, allowing for meaningful comparisons between Chinese and Ghanaian adolescents.

*Structural relationships (path analysis)*.

Hypothesis 2 hypothesized that psychological resilience, emotion regulation, academic self‐efficacy, and perceived social support would be positively and significantly associated within each cultural group, but the strength and direction of the relationships would differ between Chinese and Ghanaian adolescents. After confirming measurement invariance, the researcher performed structural path analysis to assess the relationships among the latent variables in both cultural groups. This analysis allowed us to test the hypothesized associations between the constructs and compare the strength and direction of these relationships across the two groups.

As depicted in Table 4, the results of the structural path analysis supported this hypothesis, showing positive and significant relationships between the constructs in both China and Ghana. However, the strength of these relationships differed between the two groups. For example, the relationship between psychological resilience and emotion regulation was stronger in China (path coefficient = 0.45) compared to Ghana (path coefficient = 0.33), with a significant *p* value of 0.002 for China. Similarly, the relationship between emotion regulation and academic self‐efficacy was stronger in China (0.48) than in Ghana (0.39), with a significant *p* value of 0.003 for China. The relationship between academic self‐efficacy and perceived social support was also stronger in China (0.52) compared to Ghana (0.40), with a *p* value of 0.001 for China. The relationship between psychological resilience and academic self‐efficacy was found to be significant in both groups, with a stronger path coefficient in China (0.36) compared to Ghana (0.28), and a *p* value of 0.022 for China. Finally, emotion regulation and perceived social support showed significant relationships in both groups, with stronger coefficients in China (0.41) compared to Ghana (0.29), with a *p* value of 0.015 for China. These results suggest that while all relationships between the constructs were significant in both cultural groups, the strength of these relationships was generally stronger in China than in Ghana.

*Mean differences (cultural comparison)*.

Hypothesis 3 proposed that there would be statistically significant differences in the mean levels of psychological resilience, emotion regulation, academic self‐efficacy, and perceived social support between Chinese and Ghanaian adolescents. To test this hypothesis, the researcher conducted multigroup comparisons of the means for each latent variable. This involved testing the equality of means across the two groups using the results from the scalar invariance model.

Results from the multigroup mean comparisons in Table 5 reveal consistent and statistically significant differences between Chinese and Ghanaian adolescents across psychological, cultural, and socioeconomic constructs. Chinese adolescents scored significantly higher in resilience (*M* = 3.80 vs. 3.50; *t*(298) = 2.91, *p* = 0.004, *d* = 0.55, *η* ^2^ = 0.07), emotion regulation (*M* = 3.90 vs. 3.70; *t*(298) = 2.33, *p* = 0.020, *d* = 0.42, *η* ^2^ = 0.05), academic self‐efficacy (*M* = 4.10 vs. 3.90; *t*(298) = 2.48, *p* = 0.014, *d* = 0.44, *η* ^2^ = 0.06), and perceived social support (*M* = 4.00 vs. 3.80; *t*(298) = 2.63, *p* = 0.009, *d* = 0.46, *η* ^2^ = 0.06). These findings suggest moderately higher psychological and academic functioning among Chinese adolescents. Regarding cultural orientations, large and significant differences emerged. Ghanaian adolescents reported stronger collectivist values (*M* = 3.50 vs. 2.10; *t*(298) = 11.25, *p* < 0.001, *d* = 2.05, *η* ^2^ = 0.35), higher power distance (*M* = 3.20 vs. 2.45; *t*(298) = 6.13, *p* < 0.001, *d* = 1.20, *η* ^2^ = 0.25), and greater familism (*M* = 3.85 vs. 3.10; *t*(298) = 6.18, *p* < 0.001, *d* = 1.18, *η* ^2^ = 0.24). Conversely, Chinese adolescents scored higher in uncertainty avoidance (*M* = 3.25 vs. 2.80; *t*(298) = 2.70, *p* = 0.008, *d* = 0.48, *η* ^2^ = 0.06), suggesting a preference for structured and predictable environments. Socioeconomic indicators also favored Chinese adolescents, who reported higher subjective SES (*M* = 3.85 vs. 3.40; *t*(298) = 3.21, *p* = 0.002, *d* = 0.60, *η* ^2^ = 0.08) and regional SES/education index (*M* = 0.78 vs. 0.55; *t*(298) = 5.47, *p* < 0.001, *d* = 1.10, *η* ^2^ = 0.22). Finally, peer support (*M* = 3.95 vs. 3.70; *t*(298) = 2.45, *p* = 0.015) and school climate (*M* = 4.05 vs. 3.80; *t*(298) = 2.61, *p* = 0.010) were moderately higher in the Chinese group. In all, these results indicate that Chinese adolescents exhibit stronger resilience, self‐efficacy, emotional control, and access to supportive environments, whereas Ghanaian adolescents emphasize collectivist values, respect for authority, and family obligations. The largest between‐group differences were observed in cultural values and socioeconomic indicators, suggesting that both cultural orientation and material context significantly shape adolescent psychological and academic outcomes.

Results from the multigroup ANOVA (Table 6) indicate significant cross‐national and within‐country (urban–rural) variations across psychological, cultural, and socioeconomic constructs. For psychological resilience, urban Chinese adolescents scored the highest (*M* = 3.90, SE = 0.05), followed by rural Chinese (*M* = 3.65, SE = 0.06), urban Ghanaians (*M* = 3.60, SE = 0.06), and rural Ghanaians (*M* = 3.40, SE = 0.05); *F*(3, 296) = 8.12, *p* < 0.001, *d* = 0.65, *η* ^2^ = 0.09. Similar patterns were observed for emotion regulation (urban China *M* = 4.00 vs. rural Ghana *M* = 3.65; *F* = 5.23, *p* = 0.002, *d* = 0.50, *η* ^2^ = 0.07), academic self‐efficacy (urban China *M* = 4.20 vs. rural Ghana *M* = 3.85; *F* = 4.88, *p* = 0.003, *d* = 0.48, *η* ^2^ = 0.06), and perceived social support (urban China *M* = 4.10 vs. rural Ghana *M* = 3.75; *F* = 5.77, *p* = 0.001, *d* = 0.52, *η* ^2^ = 0.07). These moderate effects indicate that both national culture and urbanization contribute meaningfully to psychological and academic development. Regarding cultural orientations, large and highly significant differences emerged. For Individualism–Collectivism, urban Chinese adolescents scored the lowest (*M* = 2.05) and rural Ghanaians the highest (*M* = 3.60); *F* = 12.10, *p* < 0.001, *d* = 2.05, *η* ^2^ = 0.35. Similarly, power distance was lower among Chinese (urban *M* = 2.40, rural *M* = 2.55) than Ghanaians (urban *M* = 3.15, rural *M* = 3.25); *F* = 11.25, *p* < 0.001, *d* = 1.20, *η* ^2^ = 0.25.

For uncertainty avoidance, Chinese adolescents scored higher (urban *M* = 3.30, rural *M* = 3.20) than Ghanaians (urban *M* = 2.85, rural *M* = 2.75); *F* = 4.65, *p* = 0.004, *d* = 0.48, *η* ^2^ = 0.06. The highest familism scores were recorded among rural Ghanaians (*M* = 3.90) followed by urban Ghanaians (*M* = 3.80); *F* = 13.40, *p* < 0.001, *d* = 1.18, *η* ^2^ = 0.24, confirming that family obligations are strongest in rural Ghanaian contexts. For socioeconomic indicators, urban Chinese adolescents reported the highest subjective SES (*M* = 3.90) and rural Ghanaians the lowest (*M* = 3.30); *F* = 6.90, *p* < 0.001, *d* = 0.65, *η* ^2^ = 0.09. The regional SES/education index showed pronounced variation: urban China (*M* = 0.80), rural China (*M* = 0.75), urban Ghana (*M* = 0.60), and rural Ghana (*M* = 0.50); *F* = 15.12, *p* < 0.001, *d* = 1.10, *η* ^2^ = 0.22 highlighting the structural advantages of Chinese and urban contexts. Finally, peer support and school climate were significantly higher among urban Chinese adolescents (peer support *M* = 4.00; school climate *M* = 4.10) compared to rural Ghanaian peers (peer support *M* = 3.70; school climate *M* = 3.75); *F*‐values ranged from 4.88 to 5.12, *p* < 0.01, *d* = 0.50, *η* ^2^ = 0.06–0.07. In all, these results indicate that urban Chinese adolescents consistently outperform all other groups across psychological, academic, and social constructs, while rural Ghanaian adolescents exhibit the strongest collectivist and familism values. The largest differences occur in cultural orientations (IC, power distance, familism) and regional SES, while psychological and school‐related constructs show moderate effects. These findings underscore that adolescent outcomes are jointly shaped by national culture and within‐country socioeconomic context, emphasizing the significance of urban–rural heterogeneity in cross‐cultural psychological research.

*Mediation effects*.

This hypothesis explored whether resilience enhances self‐efficacy indirectly by strengthening emotional regulation and perceived social support. This perspective recognizes that resilient adolescents may not only draw on internal coping strategies but also rely on external networks of support, both of which collectively reinforce their belief in their academic abilities. Examining this mediation pathway provides insight into the mechanisms that bridge resilience and academic outcomes.

The findings in Table 7 demonstrate that emotion regulation and perceived social support significantly mediate the relationship between psychological resilience and academic self‐efficacy. The direct effect of resilience on self‐efficacy was positive and significant (*B* = 0.31, *p* < 0.001), indicating that resilient students exhibit greater academic confidence even without mediators. When mediating effects were included, both emotion regulation (*B* = 0.17, *p* = 0.001; VAF = 28%) and social support (*B* = 0.13, *p* = 0.004; VAF = 20%) served as significant pathways, explaining 38%–42% of the variance in self‐efficacy. Emotion regulation emerged as the stronger single mediator, highlighting its central role in translating resilience into academic confidence. The serial mediation models provided deeper insight into the mechanisms linking resilience and self‐efficacy. The pathway resilience → emotion regulation → social support → self‐efficacy was significant (*B* = 0.08, *p* = 0.008; *R* ^2^ = 0.45; VAF = 13%), suggesting that resilience enhances emotional control, which strengthens social connections and ultimately improves self‐efficacy. The reverse sequence (resilience → social support → emotion regulation → self‐efficacy) was also significant but weaker (*B* = 0.06, *p* = 0.036; VAF = 9%). The total indirect effect (*B* = 0.29, *p* < 0.001) accounted for nearly half of the total effect (*B* = 0.60, *p* < 0.001; *R* ^2^ = 0.58), confirming that resilience influences self‐efficacy through both direct and mediated routes.

*Moderation effects*.

The hypothesis posited that the indirect effects of resilience on academic self‐efficacy through emotion regulation and perceived support are not uniform, but rather shaped by broader sociocultural conditions. Cultural norms, values, and support structures may amplify or constrain the role of these mediators, thereby influencing how resilience translates into self‐efficacy. Testing moderation allows for a deeper understanding of *when* and *for whom* resilience is most effective in promoting academic adjustment.

Findings from Table 8 indicate that cultural context significantly moderated the mediation pathways between psychological resilience and academic self‐efficacy. Resilience directly and positively predicted self‐efficacy among both Chinese (*B* = 0.34, *β* = 0.30, *p* < 0.001) and Ghanaian (*B* = 0.27, *β* = 0.24, *p* = 0.002) adolescents, with a slightly stronger but nonsignificant difference favoring Chinese students. Regarding indirect effects, emotion regulation emerged as the stronger mediator in China (*B* = 0.22, *β* = 0.18, *p* < 0.001) than in Ghana (*B* = 0.11, *β* = 0.09, *p* = 0.016), with a significant group difference (Δ = 0.11, *p* = 0.008). Similarly, the mediation via perceived social support was more robust in China (*B* = 0.16, *β* = 0.13, *p* = 0.002) than in Ghana (*B* = 0.09, *β* = 0.07, *p* = 0.031), with Δ = 0.07 (*p* = 0.021). These results confirm that Chinese adolescents gain greater self‐efficacy benefits from resilience through both emotional and social pathways. In the serial mediation models, the sequence *Resilience → Emotion Regulation → Social Support → Self‐Efficacy* was stronger in China (*B* = 0.10, *β* = 0.08, *p* = 0.005) than in Ghana (*B* = 0.05, *β* = 0.04, *p* = 0.041), with a significant group difference (Δ = 0.05, *p* = 0.033). The reverse sequence *Resilience → Social Support → Emotion Regulation → Self‐Efficacy* was significant in both groups but weaker overall and culturally nonsignificant. The total indirect effects were considerably higher among Chinese adolescents (*B* = 0.37, *β* = 0.31, *p* < 0.001) compared to their Ghanaian counterparts (*B* = 0.20, *β* = 0.17, *p* = 0.003), with Δ = 0.17 (*p* = 0.006). Finally, the total effect of resilience on academic self‐efficacy was stronger in China (*B* = 0.71, *β* = 0.61, *p* < 0.001) than in Ghana (*B* = 0.47, *β* = 0.40, *p* < 0.001), confirming a more pronounced overall association within the Chinese context. Clearly, these findings suggest that while resilience enhances academic self‐efficacy in both cultures, its effects are more strongly transmitted through emotion regulation and social support among Chinese adolescents. This pattern reflects the greater cultural emphasis on emotional discipline, social harmony, and collective perseverance in the Chinese educational context, whereas Ghanaian students appear to rely on resilience in more direct or less socially mediated ways.

## 4 Discussion

### 4.1 Measurement Invariance Across Cultures

Establishing measurement invariance across the Chinese and Ghanaian samples was a critical preliminary step to ensure that the psychological constructs under investigation—resilience, emotion regulation, academic self‐efficacy, and perceived social support—were conceptualized and interpreted in comparable ways across cultural contexts. The results confirmed configural, metric, and scalar invariance, demonstrating that the factorial structure, factor loadings, and item intercepts were equivalent for both groups. This finding implies that adolescents in China and Ghana understood and responded to the items in a conceptually similar manner, thereby permitting meaningful cross‐cultural comparisons of latent means and structural relationships. The attainment of configural invariance indicates that the basic model structure, including the pattern of factor loadings, was consistent across both cultural groups. This suggests that the four constructs shared the same underlying dimensions of meaning such as perseverance and adaptability for resilience, self‐control and emotional awareness for emotion regulation, confidence in academic capabilities for self‐efficacy, and the perception of care and assistance for social support. Establishing metric invariance further confirmed that the strength of the relationships between items and their latent constructs was equivalent across groups, supporting the comparability of associations among the variables. Finally, the confirmation of scalar invariance verified that item intercepts were statistically similar, allowing for the valid comparison of mean levels between the two cultural samples.

This methodological confirmation holds important theoretical and empirical implications. It validates that any observed differences in the mean levels or structural relationships of the constructs are genuine reflections of cultural variation rather than artifacts of measurement bias or translation effects. In other words, both Chinese and Ghanaian adolescents conceptualized resilience, emotion regulation, academic self‐efficacy, and social support in ways that are functionally equivalent, even though their expressions may differ contextually due to sociocultural influences. The findings align with prior cross‐cultural validation studies (e.g., Mullen et al. 2014; Chen et al. 2015; Kassis et al. 2023; Huang and Kou 2025), which emphasize the necessity of testing measurement invariance to ensure the psychometric soundness of instruments across diverse populations. Without such validation, cultural comparisons risk conflating true psychological differences with variations in language, response style, or cultural interpretation. Thus, the establishment of invariance not only strengthens the credibility of subsequent analyses but also contributes to the broader goal of building culturally generalizable psychological models. Moreover, the successful validation across two markedly different cultural contexts—a highly collectivist East Asian setting and a culturally hybrid African context—demonstrates the robustness and cross‐contextual relevance of these constructs. It also affirms that constructs such as resilience and academic self‐efficacy, though shaped by contextual factors, possess universal psychological cores that transcend cultural boundaries. This outcome lays a rigorous empirical foundation for exploring how these constructs interact within differing socio‐educational systems in the sections that follow.

### 4.2 Structural Relationships Among Key Constructs

The structural model revealed meaningful and theoretically consistent interconnections among resilience, emotion regulation, academic self‐efficacy, and perceived social support across both cultural groups. These constructs collectively represent critical components of adolescents' adaptive functioning and learning motivation, yet their interrelations appear to be culturally nuanced. In both the Chinese and Ghanaian samples, resilience and emotion regulation emerged as strong predictors of academic self‐efficacy, indicating that the ability to manage emotions and recover from setbacks underpins students' confidence in their academic capabilities. However, the magnitude and direction of these associations varied considerably across contexts, reflecting different sociocultural systems of meaning and value orientation. Consistent with socio‐cultural and self‐construal theories (Markus and Kitayama 1991; Gross and John 2003; Kassis et al. 2023), Chinese adolescents exhibited stronger interrelationships among the constructs particularly between resilience and emotion regulation, and between emotion regulation and academic self‐efficacy. These findings underscore how emotional restraint, perseverance, and self‐discipline, which are deeply embedded in collectivist and Confucian traditions, function as key psychological mechanisms for academic success. In this context, emotion regulation serves not only as a personal coping strategy but also as a social expectation aligned with maintaining harmony, fulfilling parental aspirations, and achieving academic excellence. The strong path coefficients suggest that adolescents in such environments internalize cultural scripts that link controlled emotional expression with persistence and scholastic confidence. In contrast, the relatively weaker structural paths among Ghanaian adolescents may reflect differences in cultural socialization, educational structures, and environmental constraints. Ghanaian adolescents operate within a sociocultural milieu that blends communal values with emerging individualistic tendencies. However, limited access to psychosocial resources, inconsistent mentoring, and less institutional emphasis on emotional development in schools may reduce the reinforcement of connections among these constructs (Huang et al. 2025; Kassis et al. 2023). Moreover, cultural norms in Ghana often allow for more expressive emotional behavior, which may moderate the direct role of emotion regulation in building resilience or academic self‐efficacy. Thus, while Ghanaian adolescents demonstrate adaptive potential, the systemic and contextual conditions in which they develop may attenuate the psychological integration seen in more structured educational and familial contexts. In all, these results illustrate that while resilience and emotion regulation universally predict academic self‐efficacy, the strength, direction, and manifestation of these relationships are influenced by cultural models of emotion, motivation, and support. The findings highlight the importance of situating psychological constructs within the broader social and cultural frameworks that give them meaning. For researchers and educators, this implies that interventions designed to enhance students' resilience or academic self‐efficacy should be contextually grounded, taking into account the cultural expectations, educational policies, and socialization patterns that shape adolescents' emotional and motivational development.

### 4.3 Cross‐Cultural Mean Differences

The observed higher mean levels of resilience, emotion regulation, self‐efficacy, and perceived social support among Chinese adolescents point to the influence of deep‐rooted cultural systems that emphasize emotional restraint, social harmony, and academic perseverance. In Chinese collectivist contexts, children are socialized from an early age to value group cohesion, filial piety, and perseverance in the face of adversity—attributes that directly reinforce adaptive coping and resilience (Chen et al. 2015; Gross and John 2003; Kassis et al. 2023). Emotional regulation, for instance, is not merely a personal trait but a culturally endorsed strategy to maintain social order and avoid interpersonal conflict. Similarly, the high levels of perceived social support may stem from the strong interdependence among family members and peers, as well as the supportive educational structures that encourage collaboration and academic achievement (Ansong et al. 2019). By contrast, the relatively lower mean scores among Ghanaian adolescents may be attributed to contextual and structural factors rather than inherent psychological limitations. Ghana's educational and social systems often face challenges such as limited access to psychosocial resources, overcrowded classrooms, and inadequate counseling services, which can affect students' perceived efficacy and emotional adjustment (Ansong et al. 2019). Additionally, cultural orientations in Ghana tend to blend collectivist and individualist tendencies where communal support exists but may not always translate into consistent institutional or parental involvement. These contextual disparities suggest that differences in adolescent well‐being are intertwined with broader socioeconomic and educational conditions (Okocha et al. 2025). The findings, therefore, underscore that academic and emotional functioning cannot be fully understood in isolation from the cultural and institutional frameworks that shape adolescents' development. They highlight the need for culturally sensitive interpretations of psychological constructs, as resilience and self‐efficacy may manifest differently across societies with distinct value systems. In essence, the cross‐cultural mean differences observed between Chinese and Ghanaian adolescents illuminate the powerful role of sociocultural context in shaping how young people perceive challenges, regulate emotions, and access social support within their educational environments.

### 4.4 Mediating Role of Emotion Regulation and Social Support

Emotion regulation and perceived social support emerged as pivotal mediating mechanisms linking resilience to academic self‐efficacy across both cultural groups. The findings indicate that resilient adolescents who possess the capacity to manage their emotions effectively and who perceive strong social backing from family, peers, and teachers are more likely to exhibit heightened academic confidence and persistence. This dual mediation underscores the intertwined roles of internal coping mechanisms and external support systems in fostering adaptive academic functioning. From an ecological and developmental resilience perspective (Kaya et al. 2024; Xu and Xu 2025; Gross and John 2003; Kassis et al. 2023), resilience is not merely an inherent psychological attribute but a dynamic process that unfolds through continuous interactions between the individual and their environment. Within this framework, emotion regulation serves as a critical intrapersonal mediator transforming the emotional consequences of stress and challenge into constructive motivation and cognitive focus. Adolescents who can modulate negative emotions, such as anxiety or frustration, are better equipped to maintain self‐efficacy beliefs, sustain academic effort, and recover from academic setbacks. Thus, emotion regulation functions as the internal bridge through which resilience translates into productive academic engagement. Simultaneously, perceived social support acts as an interpersonal mediator that reinforces and sustains the effects of resilience. Supportive relationships provide adolescents with a sense of belonging, reassurance, and external validation that strengthen their belief in their academic capabilities. Teachers, parents, and peers who model encouragement and empathy create an enabling context that allows resilient tendencies to manifest more effectively in academic domains. The perception that one is valued and supported by others fosters optimism and reduces the psychological burden associated with academic stress, ultimately enhancing self‐efficacy.

The combined influence of emotion regulation and social support suggests that academic self‐efficacy emerges through a synergistic interaction between internal emotional adaptability and external relational stability. This finding aligns with social‐cognitive theory, which posits that self‐beliefs are shaped through reciprocal interactions between personal and environmental factors (Bandura 1997). It also reinforces the notion within ecological resilience theory that optimal adaptation is achieved when individual competencies are complemented by accessible and nurturing social systems. Furthermore, the mediating roles may differ in strength and form across cultural contexts. In collectivist cultures, such as China, perceived social support may exert a stronger mediating influence due to the cultural emphasis on interdependence and relational harmony (Kaya et al. 2024). Conversely, in Ghana, emotion regulation may play a relatively more prominent role given the increasing importance of self‐directed coping in environments with limited institutional or parental scaffolding. This variation highlights the importance of understanding resilience processes as culturally embedded rather than universally uniform. In sum, the findings demonstrate that the development of academic self‐efficacy is not solely an outcome of personal resilience but rather a product of the dynamic interplay between emotional regulation and perceived social resources. Strengthening both domains through interventions that cultivate emotional intelligence and promote supportive school and family environments can enhance students' capacity to thrive academically despite adversity.

### 4.5 Cultural Moderation of the Mediation Pathways

Cultural context significantly moderated these indirect pathways, with effects notably stronger among Chinese adolescents. In Confucian‐influenced societies, where emotional discipline and interdependence are culturally endorsed, emotion regulation and social support function as powerful transmitters of resilience to self‐efficacy (Kassis et al. 2023). By contrast, the weaker mediation observed in Ghana may be attributed to limited formal support systems and larger structural barriers within schools (Hanımoğlu 2025; Ntumi et al. 2025). Nonetheless, resilience among Ghanaian adolescents may operate through alternative mechanisms such as personal agency, community ties, and spiritual coping, reflecting culturally embedded adaptive resources. Overall, these moderation findings affirm that while the pathways between resilience, emotion regulation, and self‐efficacy are universal in direction, their intensity and structure are shaped by sociocultural realities.

The findings of the study provide important theoretical insights into the cross‐cultural dynamics of resilience, emotion regulation, social support, and academic self‐efficacy. First, the evidence of measurement invariance across Chinese and Ghanaian adolescents strengthens the theoretical validity of these constructs as universal psychological resources. This suggests that resilience, emotion regulation, social support, and self‐efficacy can be conceptualized and measured consistently across diverse cultural settings, supporting theories that emphasize the universality of core psychological processes while acknowledging contextual variations. Second, the structural associations among resilience, emotion regulation, perceived social support, and academic self‐efficacy reinforce resilience theory and social‐cognitive theory by confirming that resilience operates as a protective factor that enhances adaptive functioning. The differential strength of relationships across cultural contexts highlights the role of culture in shaping developmental processes, aligning with ecological and cultural psychology theories that underscore the embeddedness of psychological processes within sociocultural systems.

Third, the observed mean differences between Chinese and Ghanaian adolescents extend theoretical debates about cultural influences on psychological resources. These differences suggest that collectivist versus collectivist‐but‐resource‐constrained contexts may shape resilience, regulation strategies, and perceptions of support differently, thereby challenging the assumption of cultural uniformity in psychological outcomes (Ansong et al. 2019).

Fourth, the mediation results provide empirical support for resilience and social support frameworks by demonstrating that resilience enhances academic self‐efficacy not only directly but also indirectly through emotion regulation and perceived support. This highlights the multi‐layered pathways through which resilience functions, extending theoretical perspectives on resilience from a static trait to a dynamic process that operates through proximal mechanisms such as regulatory skills and relational resources. Finally, the moderated mediation underscores the theoretical importance of cultural context in resilience processes. The stronger indirect effects observed among Chinese adolescents suggest that in structured and collectivist environments, resilience more readily translates into enhanced regulation and perceived support, which in turn boosts self‐efficacy. In contrast, the weaker pathways in the Ghanaian context point to the influence of resource limitations and structural challenges on these theoretical models. This advances cross‐cultural resilience theory by showing that while the mechanisms of resilience may be universal, their effectiveness is contingent on cultural and structural affordances.

The findings have significant implications for educational practice and policy in both China and Ghana. The strong relationships between psychological resilience, emotion regulation, and academic self‐efficacy in China suggest that interventions aimed at improving academic performance might benefit from focusing on emotional regulation skills. In contrast, in Ghana, where these relationships are comparatively weaker, interventions may need to focus on bolstering academic self‐efficacy through targeted support and resource provision, particularly in under‐resourced schools. Furthermore, the results underscore the need for culturally responsive intervention programs. In China, where social support tends to be formalized within family and academic institutions, strengthening family–school partnerships and teacher mentoring programs may prove most effective. In Ghana, however, community‐based and peer‐support models that leverage social cohesion and extended family systems may provide greater benefits for enhancing adolescents' perceived social support and self‐efficacy. For policymakers, the results highlight the importance of contextualizing resilience‐building frameworks within existing educational infrastructures (Ansong et al. 2019). National education strategies should integrate socio‐emotional learning components that promote emotion regulation and adaptive coping. Schools can also incorporate resilience training modules into guidance and counseling programs to help students manage academic stress and maintain self‐efficacy.

Notwithstanding its strong findings, the study has some limitations. First, the use of self‐report questionnaires may have introduced social desirability and response biases, though anonymity and the use of validated, culturally adapted instruments helped mitigate these effects. Second, the sample's geographical coverage, particularly in China, was not fully representative of the countries' regional and socioeconomic diversity, limiting generalizability. Future studies should employ stratified sampling to better capture regional variations. Third, the cross‐sectional design restricts causal inference; longitudinal studies are needed to track how resilience, emotion regulation, social support, and self‐efficacy evolve over time. Lastly, the study's focus on only two cultural contexts constrains broader applicability. Expanding to more countries and incorporating mixed‐methods approaches combining quantitative surveys with qualitative interviews would yield deeper insights into the mechanisms connecting these psychological constructs.

## 5 Conclusion

This study provides valuable insights into the cross‐cultural dynamics of psychological resilience, emotion regulation, academic self‐efficacy, and perceived social support among Chinese and Ghanaian adolescents. While these constructs demonstrated universal relevance, their interrelationships varied in strength and pattern across the two contexts, reflecting the influence of sociocultural and structural factors. Among Chinese adolescents, the stronger associations between resilience, emotion regulation, and self‐efficacy underscore the role of emotional discipline and social cohesion embedded in collectivist traditions. In contrast, the relatively weaker relationships in Ghana highlight the challenges posed by limited educational resources and less formalized support structures. These findings affirm that while resilience processes may be universal, their expression and effectiveness are shaped by cultural and contextual realities. The study makes several important contributions to theory and practice. Methodologically, it confirms measurement invariance of key psychological constructs across two non‐Western contexts, ensuring that observed differences reflect genuine cultural variation. Theoretically, it extends resilience and social‐cognitive frameworks by elucidating the mediating roles of emotion regulation and social support and demonstrating their culturally contingent nature. Practically, the results call for culturally responsive interventions emphasizing emotional regulation and family–school partnerships in China, and community‐based support and resource enhancement in Ghana. Overall, the study advances cross‐cultural understanding of adolescent development and provides an empirical foundation for designing context‐sensitive educational and psychological interventions that promote resilience and academic success globally.

## 6 Recommendations

Based on the findings of this study, it is clear that cultural contexts play a significant role in shaping the relationships between psychological resilience, emotion regulation, academic self‐efficacy, and perceived social support among adolescents. To enhance the effectiveness of psychological and educational interventions in both China and Ghana, it is crucial to implement contextually relevant programs that are aligned with the cultural and societal values of each group. In China, where emotional regulation and academic achievement are closely intertwined, interventions could focus on enhancing emotional intelligence skills alongside academic resilience training. Schools should provide students with resources to better manage academic stress, promote mindfulness practices, and encourage a balanced approach to learning. Additionally, given the importance of familial and peer support in China, intervention programs could aim to strengthen the involvement of family members in fostering students' academic self‐efficacy and emotional regulation, thus reinforcing the collectivist value of mutual support.

In contrast, in Ghana, where adolescents may face different socio‐economic and educational challenges, interventions should focus on building formal and informal support networks within schools and communities. Programs that foster peer support, mentorship, and community involvement can play a key role in strengthening social support and academic self‐efficacy. Given the less structured educational system in some parts of Ghana, efforts should also be directed toward improving access to educational resources and emotional health services. Teacher training programs could emphasize culturally responsive pedagogies that take into account the unique challenges faced by Ghanaian adolescents, including socio‐economic pressures and limited access to educational resources. By empowering educators to integrate strategies that promote emotional regulation and academic resilience, these programs could foster an environment that nurtures students' psychological well‐being and academic success. Additionally, given the importance of community in Ghanaian culture, incorporating community leaders and elders into educational initiatives can further support students' development by offering guidance and reinforcing the value of resilience and emotional well‐being. These recommendations underline the importance of culturally tailored interventions in addressing the unique developmental needs of adolescents in different cultural settings. By understanding the nuances of each context, educational practitioners and policymakers can create environments that not only promote academic achievement but also support the holistic development of adolescents as they navigate the challenges of growing up.

## Funding

The author has nothing to report.

## Ethics Statement

This study received ethical clearance from the Ethical Review Committee of the University of Education, Winneba. Ethical considerations were rigorously observed, including strict measures to ensure the confidentiality of data and to safeguard the emotional well‐being of adolescent respondents.

## Consent

Informed consent was obtained from all participants, and for those under 18 years of age, parental or guardian consent was also secured. Participants were fully informed of the study's purpose, the voluntary nature of their participation, and their right to withdraw at any point without penalty.

## Conflicts of Interest

The author declares no conflicts of interest.

## Data Availability

The data that support the findings of this study are available on request from the corresponding author. The data are not publicly available due to privacy or ethical restrictions.
